# Jack of All Trades, Masters of One?

**DOI:** 10.5811/westjem.2017.10.36890

**Published:** 2017-12-05

**Authors:** Chris Merritt

**Affiliations:** Alpert Medical School of Brown University, Department of Emergency Medicine, Providence, Rhode Island. Rhode Island Hospital/Hasbro Children’s Hospital, Department of Emergency Medicine and Pediatrics, Providence, Rhode Island

“Ladies and gentlemen, this is your captain speaking. Actually I’m not a pilot; I’m an ER doctor. But in my lifetime I’ve been on over 200 flights, so I think I have a good idea of how the process works. I really love planes, and I’ve always thought it would be fun to fly one. In fact, one of my best friends is a pilot, and I’ve spent a lot of time in airports, and my grandfather was in the Air Force. So I think we should be just fine. Please sit back, enjoy the flight, and we’ll see you in Atlanta.”

No passenger in their right mind would stay on that plane. And hopefully no emergency physician in their right mind would ever say such a thing from the cockpit. And yet, we do much the same thing (albeit with less immediate risk) when we take on roles for which our only training is, essentially, that we really like planes and we’ve spent a lot of time in airports. Being emergency physicians prepares us for many things – but our skills may not translate directly into other realms.

In particular, our training and experience as clinicians may only partially prepare us to be educators. The era of “see one, do one, teach one” is as problematic when training education leaders as it is to training in clinical skills. Learning to teach emergency medicine simply by having been taught emergency medicine may not be enough. Without professional development aimed at understanding theoretical frameworks, rigorous assessment, evaluating educational programs, and formulation of answerable education research questions, the quality of the outcomes will be limited at best.

Emergency physicians are tasked with educational roles in every domain of our careers. We teach and learn from our patients, their families, our colleagues and our peers, in every realm in which we operate, whether clinical, administrative, or academic. Emergency physicians are nothing if not educators. Increasingly, though, emergency physicians are called upon as educational *leaders* and *scholars*, both within and beyond our specialty. Because emergency physicians are typically called upon to teach, lead, and discover, we must improve the quality of our educational efforts in each of these realms.

Training in education comes in many flavors. Faculty development programs have typically engaged clinician educators in ongoing skill development.^1^,^2^ Many institutions have coalesced individual faculty development offerings into varying levels of certification.^3^ Still, as roles for clinician educators have expanded to include active engagement in health professions education (HPE) innovation, dissemination and scholarship,^4^ there is an increasing need for high-quality education research in HPE.^5^ As HPE has become progressively more sophisticated, clinicians with education roles and aspirations have begun to seek opportunities for more advanced training. In response to this demand, the number of certificate and graduate-level programs in HPE has risen nearly exponentially. Where in the mid-1990s there were fewer than 10 masters degree programs in HPE, the number has now reached well over 100 programs worldwide.^6^–^8^ The rise of online-only or asynchronous graduate degree programs has opened the doors to learners who live far from the institutions at which they study.

But why would EM clinician-educators – many of whom may already have substantial educational roles – pursue further formal training? Surely there is no requirement for masters-level or fellowship training for the emergency physician to assume scholarly or leadership roles in education. That said, evidence of educational faculty development is increasingly required of faculty worldwide. What advantages can formalized advanced training programs – fellowship programs, certificate training programs, or graduate degree programs – offer?

## FOUNDATIONAL EDUCATIONAL KNOWLEDGE

The large majority of those who complete graduate programs or fellowships in medical education report that these programs had a strong influence on their educational skills and practices.^2^,^10^,^11^ Understanding a structured, evidence-based approach to curriculum development lends itself to an improved educational “product,” as well as increasing the likelihood that educational innovation is disseminated as scholarship.^12^ Most master’s programs use applied learning approaches, which require learners to develop true-life examples of teaching practice, curriculum development, and assessment and evaluation schemes. Learning and applying what is learned in this manner lends itself to more fully-realized skill and understanding.

There exist a number of core domains within the scope of HPE training programs, including theories of teaching and learning, teaching practice (including educational methods and curriculum development), assessment and evaluation, research methods and scholarship, and leadership and management.^7^ Within each of these core content areas lies the foundational knowledge and skills felt to be necessary for leaders in education scholarship and practice. The impact seems greater than simply providing the knowledge and technical skills, however. The literature identifies a number of additional potential benefits of advanced training in education beyond technical and theoretical expertise.

Medical faculty who seek formal training in HPE report that they are more prepared and more productive than their peers who follow more traditional routes, with improved professional educational activities and increased engagement in scholarly activities.^10^ Master’s degree graduates report even greater impact, including greater ability to institute curriculum reforms and improved assessment and feedback practices, but also greater engagement in scholarly activities and a higher rate of journal publication in education scholarship.^10^ These skills in research will both advance the field as well as assist with promotion within academia.

## FROM LEARNING COMMUNITY TO COMMUNITY OF PRACTICE

Beginning in the learning communities fostered within HPE programs – via relationships with faculty, mentors, and other learners – learners begin to form larger and more interconnected personal learning networks. These can be bolstered through social media, professional societies, and other endeavors.^13^ As these learning networks become organized into formal communities of practice, they are often fruitful in developing ongoing professional partnerships within and beyond institutional boundaries.^14^ Sharing common language and interests, learned through didactic work and network-building, promotes a connection to the broader community of educators, both within and beyond emergency medicine.

### Mentorship

Perhaps the most important factor in academic productivity and success, the chance to establish and develop a mentorship relationship is among the drivers of satisfaction among graduates of advanced programs in HPE. Mentorship and collaboration build capacity for educational scholarship and research.^5^,^15^ Serving as role models, coaches, and occasionally task-masters, mentors can model the type of lifelong learning necessary to maintain competence and retain passion. As each cohort of learners matures, the community of co-learners may also play a supportive peer-mentorship role; learners help one another set goals, review progress, and maintain enthusiasm for a variety of projects.^11^ Having experienced the vitality of a successful mentoring relationship, program graduates are well positioned to begin to provide mentorship to more junior learners. Anecdotal and empirical evidence suggests that although formal mentorship roles are established for the duration of a course of study, these relationships often continue well beyond graduation.^16^

### Professional Identity Formation

Emergency physicians (and physicians and professionals of all stripes) undergo a constant evolution of identity. This evolution is punctuated by a series of milestones, from the ceremonial white coat ceremonies in medical schools to graduations marking professional advancement and formal boundaries of entrustment. But this evolution in professional identity does not end with the completion of training. Many clinician-educators move through a series of intermingled overlapping stages, identifying with various roles (physician, teacher, administrator, researcher, and leader) to greater or lesser degree depending on the influences and interactions of professional life.

A firm identity as an educator need not require formal education training, but it is clear that this professional identification is stimulated when surrounded by like-minded colleagues, performing roles increasingly central to the educator’s mission, and reinforced through shared experience, interests, and activities. As an educator’s expertise develops so does the identity as an educator. A formal training environment, emphasizing scholarship and innovation, reinforces the developing professional identities of participants, moving from *something I do* (“I teach”) to *someone I am* (“I am an educator”). Education leaders may be described as master adaptive learners, with focus on deep understanding of education theory, practice and improvement.^17^ Often described as a transformative experience, advanced HPE training can solidify this sense of purpose and commitment as an educator.^11^

## DEVELOPMENT OF AREAS OF FOCUS IN ACADEMIA AND CAREER ADVANCEMENT

While still possible to hold a position as an academic medical educator without formal qualifications, it is no longer sufficient, as Hu et al. report, “to remain the ‘enthusiastic amateur.’”^18^ Clinical or administrative expertise, once common criteria for appointment to educational leadership roles, no longer support this type of academic advancement. Particularly within medical schools, where non-clinician educators often have greater expertise and longer records of education scholarship than do clinicians, the cachet that comes with formal training may pave the way for advancement of education leaders. In addition to laying the groundwork for a program of education scholarship, helping to build professional networks, and solidifying professional identity, formal training programs in HPE may provide the type of credential that a reputation as an enthusiastic teacher may not. Due to the advanced training and credentials associated with that training graduates may be called upon more often for consultation and expertise, in turn leading to greater responsibility. Though difficult to ascribe career advancement to formalized training alone, the exposure and recognition that result may certainly contribute to the likelihood of new opportunities.

### Leadership Development

Simply by joining the community of practice of emergency medicine educators, broadening one’s own personal learning network, and achieving expertise in the discipline of HPE, leadership opportunities will arise. Leadership skills – like many of the other skills that emergency physicians learn throughout our professional lives – can be observed, taught, and learned. Most advanced training programs in HPE contain a dedicated leadership component; leadership and management are included in the core content of most master’s degree programs in HPE.^7^ By focusing a significant portion of their curriculum on explicit understanding of leadership models, organizational structure, strategic management, and conflict resolution, these programs prepare graduates to be not just educators, but educational leaders. These leadership skills are broadly applicable, though inconsistently taught during clinical training. Leadership skills are valued by HPE training program participants and their employers, and have been reported to have significant impact on attitudes, knowledge, skills and behavior.^19^

## CONCLUSION

Emergency physicians are well situated to move into leadership roles in education and education scholarship. As HPE has become increasingly professionalized, and the demands for rigor in education scholarship grow, emergency physicians are likely to continue to seek opportunities for formalized advanced training in education and education scholarship. As these advanced programs increase in number and scope, those clinicians who seek to further develop into leaders and clinician educators will likely increasingly be expected to attain such expertise.

Though the literature examining the impact of advanced programs in health professions education is in its infancy, early evidence suggests real value for their graduates. Graduates report gaining much more than technical teaching skills, though these are clearly crucial to building the foundation in education practice, leadership and scholarship. Participation in a program of advanced training helps shepherd the learner into the community of practice of medical educators, catalyzing connections in an ever-expanding network of collaborators and colleagues. Formal HPE programs can align learners with mentors whose guidance is crucial in developing skills and capacity as well as networking and career direction. Graduates of HPE programs report greater confidence and self-efficacy, as well as a more well-defined sense of professional identity. Formally-trained educators appreciate the effect that a “credential” has on career advancement, while recognizing that it takes more than a diploma to achieve success as a clinician educator; a well-developed personal learning network and community of practice are important for longevity.

Programs of faculty development in HPE are not without costs, both real financial costs and opportunity costs. Commitment to a longitudinal course of study in education may preclude learners from other opportunities, and protected time to fully engage in a learning program is a scarce commodity. Tuition for graduate degree programs may be costly, and if borne by the learner alone may or may not yield an acceptable return on investment. The decision to pursue HPE training, as well as which specific type of program to pursue (interleaved faculty development, post-graduate fellowship, certificate programs or degree-granting graduate program) is a choice best left to the learner. Factors including institutional support, protected time, location, mentorship, and other considerations are unique to each individual, and there is likely no universal “best fit.” Working professionals must be able to find a balance of time committed to their program and time committed to their other personal and professional lives. Master’s programs may require two to three years or longer to complete, and though criteria exist to ensure that programs adhere to the highest standards, this may be difficult to assess from the perspective of the prospective learner.^20^,^21^ These are real considerations when embarking on such a program of study.

Though emergency physicians pride themselves on being able to do or teach nearly anything at any time, the pressures in academic practice often push faculty toward increasing specialization. From enhancing technical teaching skills to preparing working professionals to pursue careers as clinician educators, education leaders, and education scholars, advanced degree programs in health professions education may be appealing to emergency physicians who see themselves as embodying the full role of clinician-educators. More than being engaging teachers, leaders in education must understand the processes of curriculum design, must be able to teach skills in lifelong learning, must understand programs of assessment and evaluation, and must be able to transform this work into scholarship. Education leaders have the responsibility to do these things. With mastery of these skills, the clinician educator is now able to take a seat in the cockpit to safely guide the airline, aircraft, and passengers.
